# The Impact of *BRAF V600E* Mutation Allele Frequency on the Histopathological Characteristics of Thyroid Cancer

**DOI:** 10.3390/cancers16010113

**Published:** 2023-12-25

**Authors:** Mawaddah Abdulhaleem, Saruchi Bandargal, Marc Philippe Pusztaszeri, Mohannad Rajab, Hannah Greenspoon, Joshua Ross Krasner, Sabrina Daniela Da Silva, Véronique-Isabelle Forest, Richard J. Payne

**Affiliations:** 1 Department of Otolaryngology–Head and Neck Surgery, McGill University, Royal Victoria Hospital, Montreal, QC H4A 3J1, Canada; 2 Department of Otolaryngology–Head and Neck Surgery, McGill University, Jewish General Hospital, Montreal, QC H3T 1E2, Canada; 3 Faculty of Medicine, McGill University, Montreal, QC H3G 2M1, Canada; 4 Department of Pathology, McGill University, Jewish General Hospital, Montreal, QC H3T 1E2, Canada; 5 Department of Otolaryngology—Head and Neck Surgery, King Faisal Specialist Hospital & Research Center, Al Madinah Al Munawwarah 42523, Saudi Arabia; 6 Faculty of Arts and Science, Queen’s University, Kingston, ON K7L 3N6, Canada; 7 Faculty of Medicine, Israel Institute of Technology, Haifa 3200003, Israel

**Keywords:** thyroid cancer, molecular mutations, molecular testing, *BRAF V600E*, allele frequency

## Abstract

**Simple Summary:**

This study aimed to investigate the relationship between the allele frequency (AF) of the *BRAF V600E* mutation and the histopathological features of papillary thyroid cancer (PTC), with a focus on its aggressive behavior. The research involved a retrospective chart review of 44 patients with *BRAF V600E*-positive thyroid malignancies, and the results indicated a direct correlation between *BRAF V600E* AF and aggressive histopathological behavior. Specifically, nodules with aggressive PTC features exhibited a significantly higher mean AF (25.8%) compared to the non-aggressive group (10.25%). Additionally, a significant difference in mean AF was observed between patients with positive sentinel lymph nodes (29%) and those with negative sentinel lymph nodes (17.8%). Although different histopathological subtypes showed varying mean AF values, they did not exhibit a statistically significant relationship. The study findings suggest that the *BRAF V600E* mutation, in combination with AF, can serve as a pre-operative indicator to help thyroid specialists determine the extent of thyroidectomy and the necessity of lymph node dissection, providing valuable insights for the management of thyroid malignancies in clinical practice.

**Abstract:**

Background: A *BRAF V600E* mutation in papillary thyroid cancer (PTC) has been shown to be associated with aggressive behavior. Nevertheless, not all *BRAF V600E* PTCs behave aggressively. Allele frequency (AF) is the number of mutated molecules divided by the total number of wild-type molecules at a specific location in the genome. The relationship between *BRAF V600E* AF and the histopathological features of thyroid malignancies is not well understood. We hypothesized that the *BRAF V600E* AF will correlate directly with aggressive histopathological behavior. The aim of this study was to examine this relationship. Methods: A retrospective chart review was performed for patients treated for *BRAF V600E* thyroid malignancies from 2019 to 2022 at McGill University tertiary care hospitals (*n* = 317). Patients with *BRAF V600E*-positive malignancies that included information on AF were included (*n* = 44). The correlation between AF and tumor histopathological features was analyzed. Results: Out of the 44 nodules with a *BRAF V600E* mutation, those with aggressive features of PTC had a mean AF of 25.8%, which was significantly higher than the non-aggressive group with a mean AF of 10.25% (*p* = 0.020). Additionally, there was a statistically significant difference in mean AF between patients with a positive sentinel LN (29%) and those with a negative sentinel LN (17.8%) (*p* = 0.021). Classical PTC was present in 29.5% (13/44) of nodules, with a mean AF of 15.6%. The tall cell subtype was found in 64% (28/44) of nodules, with a mean AF of 23%. Solid and hobnail subtypes were less common in this study, and there was no statistically significant relationship between AF and histopathological subtypes (*p* = 0.107). Nodules smaller than 1cm had a mean AF of 13.3%, while nodules ranging from 1 2cm had a mean AF of 20.6%, and those larger than 2cm had a mean AF of 27.7%. However, no statistical difference was observed between AF and nodule size (*p* = 0.160). Conclusion: In this study, *BRAF V600E* mutations in conjunction with AF help to determine whether thyroid malignancies will display aggressive behavior. This pre-operative finding can help thyroid specialists to determine the extent of thyroidectomy and whether lymph node dissection is required.

## 1. Introduction

Thyroid cancer rates have been increasing in incidence in the last several decades [1]. This increase is partly due to advancements in diagnostic imaging modalities allowing for early detection of small nodules. Accordingly, the likelihood of malignancy in all thyroid nodules regardless of size remains low (<10%) [2,3]. 

Genetic alterations in thyroid cancer have been well characterized in the literature, with hundreds of genetic driver mutations having been identified, the most common ones being *BRAF V600E* and *RAS* point mutations. Molecular testing was developed to better understand the nature of thyroid nodules, avoid diagnostic surgery in benign nodules, and to provide individualized care to patients. This was achieved by identifying somatic mutations in thyroid nodules and their associated likelihood of malignancy. Initially, the tests were used to help determine the optimal clinical management of Bethesda III and IV nodules by avoiding surgery in benign thyroid nodules. More recently, studies have demonstrated that these tests can also help determine the optimal extent of thyroid surgery in Bethesda V and VI nodules [4,5].

*BRAF V600E* is the most common mutation in PTC [6]. It can be found in 45–80% of classical subtypes of PTCs and 60–95% of tall cell subtypes of PTCs [7]. *BRAF V600E* mutations have been shown to be a sensitive but not specific marker for aggressive thyroid cancers, as they are more likely to be associated with aggressive histopathological features such as capsular invasion, extrathyroidal extension, and nodal metastasis [8,9]. In contrast, several studies did not report any association between this mutation and aggressive histopathological features of PTCs [10,11]. Allele frequency (AF) is the number of mutant molecules divided by the total number of wild-type molecules at a specific location in the genome. Next-generation sequencing allows for the simultaneous sequencing of millions of DNA fragments, indicating the proportion of DNA or RNA molecules in the sample that carry a particular mutation, which corresponds to the AF. AF determination is a quantitative measure used in genetic analysis to assess the proportion of a specific allele (gene variant) at a particular genomic location within a sample of genetic material. It helps to assess the relative abundance of mutated alleles within the sample. While AF focuses on the allele frequency at a specific genomic location, mean AF provides a consolidated measure, offering insights into the average allele frequency across multiple loci or within a set of samples. So, while AF provides information about the prevalence of a specific allele at a single locus, mean AF offers a summary measure, indicating the average allele frequency across multiple loci or samples. As suggested by preliminary data, it can be hypothesized that AF in part can act as a surrogate of tumor burden and, therefore, higher AFs could correlate with aggressive clinical and/or histopathological characteristics of the tumor [12]. In accordance, we hypothesized that a higher AF would be associated with aggressive histopathological features in *BRAF V600E*-mutated thyroid malignancies. The objective of this study was to explore the correlation between the *BRAF V600E* mutation AF and histopathological features of thyroid cancer.

## 2. Materials and Methods

### 2.1. Study Design, Setting, and Duration

This is a retrospective cohort study of 317 patients with thyroid nodules who underwent pre-operative molecular testing. The medical records were obtained from databases at McGill University tertiary care hospitals in Montreal, Canada, between 2017 and 2022. The study included patients who were 18 years old or older and had a thyroid malignancy with a positive isolated *BRAF V600E* mutation and pre-operative information on the AF percentage from pre-operative ThyroseqV3 molecular test from FNA samples. Patients with a *BRAF V600E* mutation in combination with other mutations, such as *TERT*, were excluded from the study. Molecular testing was performed with ThyroseqV3^®^ as this test provides information on AF, unlike other molecular tests used at our institution (Afirma^®^, ThyGenX/Thyramir^®^). 

### 2.2. Study Population and Variables

Out of 317 cases identified, 44 nodules met the inclusion criteria. Baseline characteristics were retrieved and included patient age, sex, nodule location, Bethesda score, the presence of lymph node metastasis, tumor specifics such as micro and gross extrathyroidal extension (ETE), and the longest axis measurement in centimeters (cm) of the tumor on ultrasound. Nodules were grouped by size into three categories: <1 cm, 1 to 2 cm, and >2 cm. The Bethesda system for reporting thyroid cytology was used to report the diagnoses of the fine-needle aspiration biopsy (FNA) samples [13,14]. The nodules were divided into two groups based on tumor aggressivity. Aggressive features were defined by the presence of one or more of the following: ETE, positive lymph node metastasis, and high-risk histological features (tall cell, columnar cell, hobnail/micropapillary, and diffuse sclerosing). Samples of the classical subtype with tall cell features were incorporated into the tall cell group due to its more aggressive nature compared to the regular classic subtype [15].

The final pathological sample was diagnosed based on the 2017 World Health Organization (WHO) classification of endocrine tumors [16]. The cytology and final pathology samples were evaluated by a board-certified head and neck fellowship-trained pathologist.

All participants’ personal information was coded and kept confidential and protected during data collection and analysis. Ethical approval was obtained from both McGill University Health Centre and CIUSSS West-Central Research Ethics Board in Montréal, QC, Canada (MP-05-2023-3672).

### 2.3. Statistical Analysis

The analysis was performed using Statistical Package for Social Sciences (SPSS) version 23.0. Descriptive and frequency analyses were conducted, and the distribution was expressed with frequency, mean, and standard deviation. Linear regression analyses were used to predict the relationship between AF (dependent variable) and *BRAF V600E* indicators (independent variables). The confidence interval was based on 95% and the level of statistical significance was *p*-value ≤ 0.05.

## 3. Results

### 3.1. Patient Characteristics

A total of 44 patients with *BRAF V600E*-positive thyroid nodules were included in this study. The baseline characteristics are summarized in Table 1. The average age was 45 years (±13), ranging from 19 to 75 years old. Most of the patients were females (*n* = 33, 75%). Nodules were found in the isthmus (*n* = 4, 9%), left thyroid lobe (*n* = 14, 32%), and right thyroid lobe (*n* = 26, 59%). Ultrasound-guided FNA biopsy results showed Bethesda VI nodules (*n* = 31, 71%), Bethesda V nodules (*n* = 7, 16%), and Bethesda III nodules (*n* = 6, 14%); no Bethesda IV nodules were found in this study. More than half of the patients had a positive sentinel lymph node (SLN) during surgery (*n* = 30, 68%). 

### 3.2. AF and Tumor Aggressivity 

Patients with aggressive tumors had a mean AF of 26% (±14.6), while tumors without aggressive features had a mean AF of 10% (±13.5). Higher AF was associated with aggressive features of PTC (*p* = 0.020) (Table 1 and Table 2).

### 3.3. AF and Histological Subtypes

**Figure 1:**
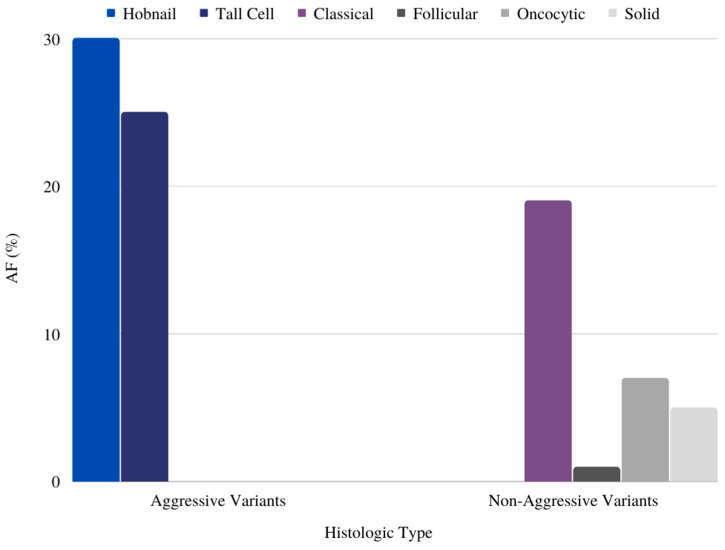
Tumor aggressiveness and AF: patients with aggressive tumors had a mean AF of 25.8%; tumors without aggressive features had a mean AF of 10.2%.

All the 44 nodules that were included were found to be malignant and diagnosed as PTC. Furthermore, the analysis identified four different histological subtypes of PTC. Classical PTC presented in 13 cases and was associated with a mean AF of 16% (±16). The tall cell subtype was present in 28 patients and was associated with a mean AF of 23% (±14). The hobnail and solid subtypes were both less common in the samples, and results showed mean AFs of 30% (±18) and 5%, respectively (Figure 1, Table 2).

### 3.4. AF and Number of Positive Central Compartment Lymph Nodes

Patients without lymph node metastasis had a mean AF of 15% (SD ± 12). Patients with ≤3 positive lymph nodes had a mean AF of 28% (±7.26). Moreover, patients with >3 positive lymph nodes had a mean AF of 25% (SD ± 15). The relationship between AF and the number of positive metastatic lymph nodes in the central neck compartment was not significant (*p* = 0.136) (Table 2).

### 3.5. AF and Sentinel Lymph Node

**Figure 2:**
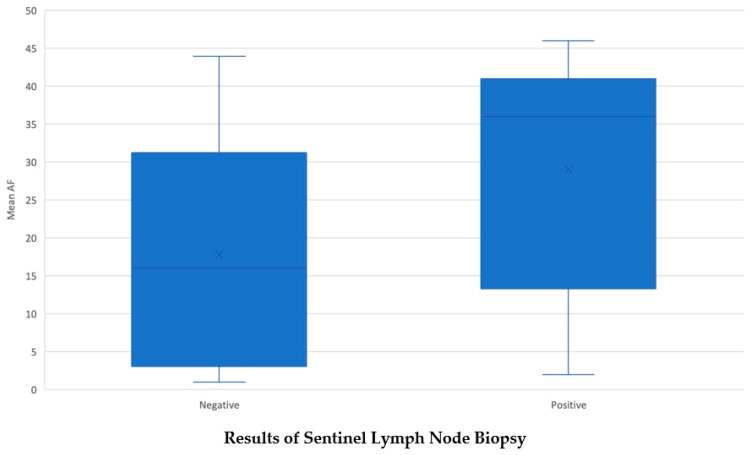
Patients with positive SLNB had higher mean AF of 29% compared to patients with negative SLNB (17.8%).

Patients with a positive SLN had a mean AF of 29% (SD ± 14.8), while the mean AF of patients with a negative SLN was 17.8% (SD ± 14.3). There was a significant statistical association between positive SLN and AF (*p* = 0.021) (Figure 2, Table 2).

### 3.6. AF and Extra Thyroidal Extension

Patients with ETE had a mean AF of 21% (SD ± 14.1), whereas patients without ETE had a mean AF of 22% (SD ± 15.6; *p* = 0.861) (Table 2). 

### 3.7. AF and Nodule Size on Ultrasound

The mean AF of nodules < 1 cm was 13% (SD ± 14.6), while nodules between 1 and 2 cm in size had a mean AF of 21% (SD ± 15.3), and patients with >2 cm nodules had a mean AF of 28% (SD ± 13.8; *p* = 0.129) (Table 2).

### 3.8. Univariate Regression Analyses of AF and Patient Characteristics

The results of our study showed a clear mean difference in AF among the SLN groups. As depicted in Table 1, patients with positive lymph nodes had a higher mean AF value of around 35%, while patients with negative lymph nodes had a mean AF of approximately 15% (*p* = 0.021). Figure 1 further revealed that the group with aggressive features had a significantly higher mean AF (26%) compared to the non-aggressive group (10%) (*p* = 0.020). Notably, we observed that females had a slightly higher mean AF value of 22% (SD ± 15.4) than males, with a mean AF of 19% (SD ± 15), although this difference was not statistically significant (*p* = 0.588) (Table 2). 

## 4. Discussion

PTC is the most prevalent type of thyroid cancer, accounting for as much as 85% of all thyroid cancer cases [17]. PTC can manifest in different subtypes, including classical, follicular, oncocytic, solid, tall cell, and hobnail. The tall cell and hobnail subtypes are known to be associated with a worse prognosis when compared to other subtypes [16]. Molecular testing such as ThyroseqV3^®^ has been increasingly used to provide guidance to healthcare providers on the extent of surgery required to prevent completion thyroidectomies and unnecessary total thyroidectomies, and to identify patients who might benefit from molecular-targeted therapies [17]. *BRAF V600E* is the most common mutation found in PTCs [18]. It is typically associated with more aggressive tumors with an intermediate risk of recurrence, those that are >1 cm according to the 2015 American Thyroid Association (ATA) guidelines [19,20]. Therefore, when a *BRAF V600E* mutation is detected in tumors larger than 1cm, a total thyroidectomy is preferred, regardless of other tumor features [21]. According to other studies, tumor behavior may not only depend on the detection of the *BRAF V600E* mutation but also on the AF of the mutation itself [12,22]. 

One of the key findings of our study was the significant association between high AF and the presence of aggressive features of PTC (*p* = 0.020). This finding provides further evidence that supports the hypothesis that AF may play a role in the behavior and aggressiveness of *BRAF V600E* tumors. Identifying cases of *BRAF V600E* with high AF levels may serve as a useful indicator in identifying patients who are at a higher risk of developing aggressive disease. This, in turn, may prompt the consideration of more aggressive treatment strategies aimed at improving patient outcomes.

Sentinel lymph node biopsy, which is commonly used to detect lymph node metastasis, was performed on all patients during surgery. Our analysis revealed a strong association between high *BRAF V600E* AF and a positive sentinel lymph node (*p* = 0.021), indicating that patients with high AF are more likely to have local lymph node metastasis. It has been suggested that there is no significant relationship between *BRAF V600E* mutation and lymph node metastasis, but our results suggest that this may be due to variations in AF across tumors. Specifically, *BRAF V600E* tumors with lower AFs may be less likely to metastasize to lymph nodes. 

It is well established that the size of the thyroid nodule is associated with the presence of aggressive histopathological features and distant metastasis in larger thyroid cancers [23,24]. In our study, we observed a positive correlation between *BRAF V600E* AF and nodule size as determined with ultrasound imaging, although this relationship was not statistically significant (*p* = 0.160). 

Moreover, we examined the correlation between the number of positive lymph nodes from central compartment neck dissection and AF. While we did not observe a statistically significant relationship, we did note that patients without lymph node metastasis had a mean AF of 15%, whereas patients with >3 positive lymph nodes had a mean AF of 25%. Although this difference was not statistically significant (*p* = 0.136), it suggests a possible trend towards higher AF in cases with more extensive lymph node involvement. 

In 2018, Ahmad et al. demonstrated that ETE, which is a known marker of aggressive disease, was correlated with the *BRAF V600E* mutation [25], further supporting *BRAF V600E* as an indicator of more aggressive tumors. In our study, ETE and *BRAF V600E* mutation AF were not found to be associated (*p* = 0.861). Therefore, these data do not support AF as a predictor of ETE in *BRAF V600E*-positive thyroid nodules. 

Recent studies found that the tall cell subtype, columnar cell subtype, and hobnail subtype are associated with more aggressive disease [12,26]. The tall cell subtype was found to be linked to ETE, extensive locoregional and distant metastasis, and a worse prognosis. Our analysis did not show any statistically significant relationship between histopathological subtypes of PTC and AF (*p* = 0.107), but a trend towards a higher AF in hobnail and tall cell variants was uncovered. 

Studies have indicated that despite being classified as a stable or stagnant cancer, patients with tumors ≤ 1 cm (i.e., papillary thyroid microcarcinomas or PMCs) may develop cervical nodal disease in approximately 10% of cases [27]. A 2019 study by Perera D. et al. investigated the genomics of PMCs with lateral neck lymph node metastases. They performed molecular testing on tumors from pN1b patients (those with at least one significant lymph node metastasis) and pN0 patients (those with at least five histologically normal lymph nodes) and found that approximately 61% of the samples tested had the *BRAF* mutation, mostly *BRAF V600E* (except for two samples). Interestingly, the study found approximately equal proportions of *BRAF* mutations in both groups, suggesting that the *BRAF* mutation may not necessarily lead to nodal disease, despite previous associations [27]. It is possible that this observation is linked to the AF of *BRAF V600E* tumors. Tumors with low AF are linked to pN0 patients, while those with higher AF are associated with pN1b patients and more aggressive disease. This observation is supported by a recent analysis conducted on a Chinese population with *BRAF* thyroid tumors, which revealed a linear increase in the risk of advanced T and N stages as the *BRAF* allele frequency increases [28]. Importantly, our analysis also found that patients with pN1a had a higher AF compared to pN0 patients. 

This study had some limitations, such as its retrospective design and the potential for geographic selection bias due to the dual-center approach. Additionally, pathologists and surgeons were not blinded to the AF results. To address these limitations, future studies could consider using a multi-center, prospective, blinded model and larger sample size. 

## 5. Conclusions

To summarize, this study demonstrated a significant association between *BRAF V600E* AF and aggressive histopathological features of PTC as well as positive sentinel lymph nodes, indicating tumor spread to the central neck compartment. These results could potentially improve pre-operative decision-making by aiding in determining the necessary extent of thyroidectomy and central compartment neck dissection.

## Figures and Tables

**Table 1:** Characteristics of *BRAF V600E* patients (*n* = 44), including mean and SD.

		*n*	Frequency	AF Mean	SD
**Sex**	Female	33	75	22.1	15.4
	Male	11	25	19.2	15
**Age mean = 45 years**		44	N/A	N/A	13
**AF (%)**		N/A	N/A	21	15
**Histologic Subtype**	Classical	13	29.5	15.6	16
	Hobnail	2	4.5	30	18
	Solid	1	2	5	N/A
	Tall cell	28	64	23	14
**Positive central compartment lymph nodes**		26	59.1	N/A	N/A
	0	18	40.9	15	12
	≤3	16	31.8	28.3	7.26
	>3	10	22.7	25	15
**SLN Result**	Negative	30	68.2	17.8	14.3
	Positive	14	31.8	29	14.8
**Ultrasound Size (cm)**	<1.0	6	13.6	13.3	14.6
	1–2	27	61.4	20.6	15.3
	>2	11	25	27.7	13.8
**Site**	Isthmus	4	9.1	23	22.6
	Left lobe	14	31.8	18.3	15.9
	Right lobe	26	59.1	22.8	14.1
**FNA Result**	B3	6	13.6	15.3	12
	B5	7	15.9	15.7	13.6
	B6	31	70.5	23.8	15.8
**Presence of ETE**	Negative	36	81.8	22	15.6
	Positive	8	18.2	20.5	14.1
**Aggressive Findings**	Aggressive	36	81.8	25.8	14.6
	Not Aggressive	8	18.1	10.25	13.5

Characteristics of *BRAF V600E* patients (*n* = 44), including mean and SD; AF = allele frequency; SLN = sentinel lymph node; FNA = fine-needle aspiration; B3 = Bethesda category III; B5 = Bethesda category V; B6 = Bethesda category VI; ETE = extra thyroidal extension.

**Table 2:** Association among clinicopathological variants and AF in *BRAF V600E* patients.

Variants	95% CI	*p*-Value
**Sex**		
**Female**	16.6–27.6	0.588
**Male**	9.1–29.2	
**Site**		
**Isthmus**	−12.9–58.9	0.666
**Left lobe**	9.1–27.4	
**Right lobe**	17.1–28.5	
**Histological subtype**		
**Classical**	7.6–29.4	0.107
**Hobnail**	−135.2–195.2	
**Solid**	5.0–5.0	
**Tall cell**	19.8–30.9	
**SLN Result**		
**Negative**	12.5–23.1	0.021
**Positive**	20.5–37.6	
**ETE**		
**Negative**	16.3–26.8	0.861
**Positive**	8.7–32.3	
**Positive LNM in central compartment**	−0.472–3.358	0.136
**FNA Result**		
**B3**	2.7–27.9	0.263
**B5**	3.1–28.3	
**B6**	18–29.6	
**Ultrasound Size**		
**<1.0**	−1.9–28.6	0.160
**1.0–2.0**	18.5–37.0	
**>2.0**	14.5–26.6	
**Aggressive Findings**		
**Aggressive**	18.9–28.8	0.020
**Not Aggressive**	−1.06–21.6	

Association among clinicopathological variants and AF in *BRAF V600E* patients; AF = allele frequency; SLN = sentinel lymph node; ETE = extra thyroidal extension; LNM = lymph node metastasis; FNA = fine-needle aspiration; B3 = Bethesda category III; B5 = Bethesda category V; B6 = Bethesda category VI.

## Data Availability

The data presented in this study are available on request from the corresponding author. The data are not publicly available due to the ethics approval agreement.
